# Diabetic Renal Disease

**DOI:** 10.1155/2014/598015

**Published:** 2014-04-07

**Authors:** Stephen Fava, Samy Hadjadj, James Walker

**Affiliations:** ^1^Diabetes & Endocrine Centre, Mater Dei Hospital, Msida, Malta; ^2^Department of Medicine, University of Malta, Msida, Malta; ^3^Centre Hospitalier Universitaire Poitiers, Endocrinology, Diabetology, Poitiers, France; ^4^Université de Poitiers, UFR Médecine Pharmacie, Centre d'Investigation Clinique, Poitiers, France; ^5^CHU de Poitiers, Centre d'Investigation Clinique, Poitiers, France; ^6^Institut National de la Santé et de la Recherche Médicale, CIC1402 & U1082, Poitiers, France; ^7^NHS Lothian, University of Edinburgh, Scotland, UK

Diabetic nephropathy (DN) is an important long-term complication of diabetes. DN is now the most common cause of end-stage renal disease (ESRD) in many countries [1]. Both the increasing prevalence of type 2 DM and increased acceptance of diabetic patients into renal replacement therapy (RRT) programmes have contributed to this. Indeed, there has been a marked increase in the incidence of renal replacement therapy (RRT) for type 2 DM over time [1]. DN is also a major burden on health care budgets [2] and is associated with a reduction in health-related quality of life [3, 4]. Moreover, DN is associated with increased cardiovascular mortality in both type 1 and type 2 diabetic patients [5, 6].

Only a proportion of patients with DN progress to ESRD, and even in those who do progress, there is a long lag time between onset of DN and progression to ESRD. What is therefore needed is early diagnosis of DN and safe effective therapy which halts or slows down its progression as well as therapy which reduces the risk of adverse cardiovascular events in DN patients. The development of regenerative medicine for the cure of renal disease is another goal, which does not seem to be within our reach in the near future.

Diabetic nephropathy is associated with typical histological features. The Renal Pathology Society has proposed a histopathological classification of diabetic nephropathy [7]. It is hoped that this new classification might be useful in better staging of the disease and in stratifying risk. However, a renal biopsy is not indicated in the majority of cases.

The diagnosis of diabetic renal disease, therefore, usually relies on measuring the urinary albumin excretion rate and the glomerular filtration rate (GFR) and on exclusion of other causes of renal disease. Whilst an elevation in the urinary albumin excretion rate is often the earliest sign of DN, measurement of glomerular filtration is increasingly important as the disease progresses. Previously, serum creatinine alone was used as a maker of GFR. A long recognised problem of using serum creatinine as a measure of filtration function is that the GFR can fall to a clinically significant level before the creatinine level in the serum begins to rise—there being an inverse reciprocal relationship between serum creatinine and GFR. The laboratory measurement of serum creatinine using the Jaffe method has resulted in interlaboratory variability and more recently clinical chemistry laboratories have been restandardising assays against an isotope mass spectrometry method. Whilst serum creatinine levels are influenced by GFR, they are also related to muscle mass and other static and changing parameters. During the last decade many laboratories have reported estimated GFR (eGFR) using a variety of calculated formulae based on parameters including age, race, gender, creatinine (see above), and in some cases urea and albumin. The reporting of eGFRs was designed to identify those with modest degrees of renal impairment that may have been missed by clinicians relying on serum creatinine alone. The commonly used formulae for eGFRs, such as Cockcroft Gault and the Modification of Diet in Renal Disease (MDRD) formulae, are inaccurate at GFR levels above 60 mL/min, so many laboratories will not report specific levels above 60 mL/min and will simply report a value >60 mL/min in this cohort. There are a number of circumstances where eGFR estimates are not valid including the presence of amputations, skeletal muscle diseases, extremes of age, and extremes of weight, pregnancy, and paraplegia and rapid changes in filtration function. These are often not appreciated by clinicians reviewing results on their patients. The widespread introduction of eGFR measurement has revealed many patients with eGFRs in the 30–40 mL/min range resulting in an increased rate of referral to renal departments. Whether this has resulted in clinical benefit is debatable. How much benefit have we derived from eGFR measurements?

E. Y. Lee et al. in one of the papers in this issue have compared two formulae commonly used to estimate GFR, namely, the MDRD and the Chronic Kidney Disease Epidemiology Collaboration (CKD-EPI) in type 2 patients with DN in Korea. They conclude that the CKD-EPI formula may more accurately stratify chronic kidney disease (CKD) in those with type 2 diabetes compared to the MDRD equation. However, this finding needs to be contrasted with the findings from the paper by X. Liu et al. which investigates the performance of the various formulas used to estimate GFR. They conclude that none of the 8 formulae examined in Chinese type 2 diabetic subjects had sufficient accuracy compared to an isotopic measurement of GFR. Inspection of the Bland Altman plots in this paper starkly demonstrates the wide levels of bias and the very wide levels of agreement between the eGFRs and standard GFR. One important point that has to be stressed is that there may be racial differences in the performance of various formulas to estimate GFR.

So what should clinicians take from these and other studies investigating eGFR equations? Firstly, they are “estimates” of the GFR in the same way that serum creatinine is an estimate and adding more parameters does not necessarily increase the accuracy. Secondly, they are likely to be inaccurate if the GFR exceeds 60 mL/min. Thirdly, there are many situations where they are likely to be particularly inaccurate and this includes using a certain formula derived from one racial group being applied in a different racial cohort.

Another area of research is the search for possible biomarkers of diabetic nephropathy. These may be useful in the diagnosis or in predicting prognosis in terms of progression or of cardiovascular complications. These include the use of proteomics, new markers of renal dysfunction, and micro-RNAs. The latter are short noncoding RNAs that may have regulatory roles. In another paper appearing in this issue, R. Li et al. review the possible role of micro-RNAs in the pathogenesis of DN and as potential biomarkers.

Although blood pressure control and blockade of the renin-angiotensin-aldosterone system are well-established as effective therapies in reducing the rate of progression of diabetic renal disease, there is still a large unmet need in the developing new treatment modalities. Research has focused on finding agents which inhibit molecules or key steps in pathways thought to be important in the pathogenesis of diabetic nephropathy, such as the polyol pathway and transforming growth factor-*β*. Unfortunately, these avenues have, to date, been largely unsuccessful in providing the clinician with new therapeutic tools. In separate papers appearing in this issue, two novel therapies, namely, resveratrol (F. Xu et al.) and L-arginine (T. Claybaugh et al.), are investigated in animal models. The review paper by Li et al. appearing in this special issue also discusses the potential role of micro-RNAs as novel therapeutic modalities.

*Stephen Fava* *Stephen Fava* *Samy Hadjadj* *Samy Hadjadj* *James Walker* *James Walker*
